# 

**Published:** 1870-07

**Authors:** 


### BOOK NOTICES.

"The Guide Board to Health, Peace, and Competence; or, the Road to a happy Old Age." By W. W. Hall, M. D., Editor of *Hall's Journal of Health.* Sold only by subscription. Published by D. E. Fish & Co., Springfield, Mass. 8vo. 752 pp. $4.

"Mother come Home" is a 50 cent book, published by the National Temperance Society, 172 William St., New York, J. N. Stearnes, publishing agent; a useful book for all families, especially interesting to children.

"Country Homes." By Sereno Edwards Todd; being a practical book by a practical genius; giving full and reliable directions for choosing a home, erecting houses, out-buildings, painting, glazing, and in a way to

SAVE AND MAKE MONEY.

There are also valuable suggestions and information about domestic economy, general farming, fancy gardening, fruit raising, breeding and management of horses, cattle, sheep, swine, poultry, etc. We agree with the New York "Evening Mail," in saying that this is the most complete and comprehensive work of its kind ever published. It is profusely illustrated with plans and perspectives of buildings, and is sold by subscription only, by the HARTFORD, CONN., PUBLISHING COMPANY; J. A. STODDARD & Co., Chicago; J. D. DENISON, New York; G. P. HAWKES, Boston; J. LAWS & Co., San Francisco.

"Gray's Descriptive and Surgical Anatomy." 876 pp. 8vo. By HENRY C. LEE, Philadelphia, 1870. 462 engravings. A new American, from the fifth and enlarged English edition. Of all books of its kind, this is the most complete, full, and yet succinct; so clear, so satisfactory in all its statements, that the student as well as the experienced practitioner feels, while examining its pages, that the latest ascertained facts on all points are supplied to his hand with a clearness, a discrimination and a fairness which at once commend the volume to the confidence of the profession.

"Daeton's Physiology," published by HENRY C. LEE, Philadelphia, Pa., 695 pp. 8vo, with 274 illustrations. By Prof. DAETON, of the New York College of Physicians and Surgeons. This has for some years been a text book among medical students, and a most convenient and satisfactory book of reference for medical practitioners, as it embodies the latest results of physiological research of the most eminent men of our time, at home and abroad. It is a valuable acquisition to any medical library, especially for its stability.

"The Guide Board." A correspondent writes, June 6, 1870, "Having read many common-sense extracts from your *Journal of Health*, when the canvasser called to obtain subscriptions for 'The Guide Board to Health,' I subscribed at once, and have no occasion to feel that I did not get my money's worth.

"Job Tufton's Rest," being a story of life's struggles. By CLARA LUCAS BELFOUR. Published by the NATIONAL AMERICAN TEMPERANCE SOCIETY. 332 pp., $1.25. A most instructive volume for all, especially for every young man or woman who is under the fortunate necessity of making their own way in the world. We wish it an immense circulation.

NEW PUBLICATIONS for Sunday School Libraries.

The NATIONAL TEMPERANCE SOCIETY AND PUBLICATION

172

House have recently published the following New Books, suitable for Sunday School Libraries: —

| | |
|---|---|
| Jug-Or-Not, | $1.25 |
| The Harker Family, | 1.25 |
| Job Tufton's Rest, | 1.25 |
| Come Home, Mother, | 50 |
| Tim's Troubles, | 1.50 |
| The Drinking Fountain Stories, | 1.00 |
| Tom Blinn's Temperance Society, | 1.25 |

Address

J. N. STEARNS, *Publishing Agent,*
172 William Street, New York.

"London Lancet." Monthly, $5 a year, postage 24 cts. a year, republished promptly, by Wm. C. Hazard, 52 John St., New York, contains the latest authentic progress in medicine and surgery, throughout the world. No young physician, ambitious of usefulness and success, can afford to be without the London Lancet.

